# Traceability and Authentication of Manila Clams from North-Western Adriatic Lagoons Using C and N Stable Isotope Analysis

**DOI:** 10.3390/molecules26071859

**Published:** 2021-03-25

**Authors:** Gianluca Bianchini, Valentina Brombin, Pasquale Carlino, Enrico Mistri, Claudio Natali, Gian Marco Salani

**Affiliations:** 1 Department of Physics and Earth Sciences, University of Ferrara, 44122 Ferrara, Italy; bncglc@unife.it (G.B.); mstnrc1@unife.it (E.M.); slngmr@unife.it (G.M.S.); 2 Institute of Environmental Geology and Geoengineering of the Italian National Research Council (CNR-IGAG), 00015 Montelibretti, Italy; claudio.natali@unifi.it; 3 Elementar Italia s.r.l., Largo Guido Donegani 2, 20121 Milan, Italy; pasquale.carlino@elementar.com; 4 Department of Earth Sciences, University of Florence, 50121 Florence, Italy

**Keywords:** seafood, *Ruditapes philippinarum*, traceability, isotope ratio mass spectrometry

## Abstract

In the Adriatic lagoons of northern Italy, manila clam (*Ruditapes philippinarum*) farming provides important socio-economic returns and local clams should be registered with the Protected Designations of Origin scheme. Therefore, there is a need for the development of rapid, cost-effective tests to guarantee the origin of the product and to prevent potential fraud. In this work, an elemental analysis (EA) coupled with isotope ratio mass spectrometry (IRMS) was employed to identify the isotopic fingerprints of clams directly collected onsite in three Adriatic lagoons and bought at a local supermarket, where they exhibited certification. In particular, a multivariate analysis of C/N, δ^13^C and δ^15^N in manila clam tissues as well as δ^13^C in shells and Δ^13^C (calculated as δ^13^C_shell_–δ^13^C_tissues_) seems a promising approach for tracking the geographical origin of manila clams at the regional scale.

## 1. Introduction

**Figure 1:**
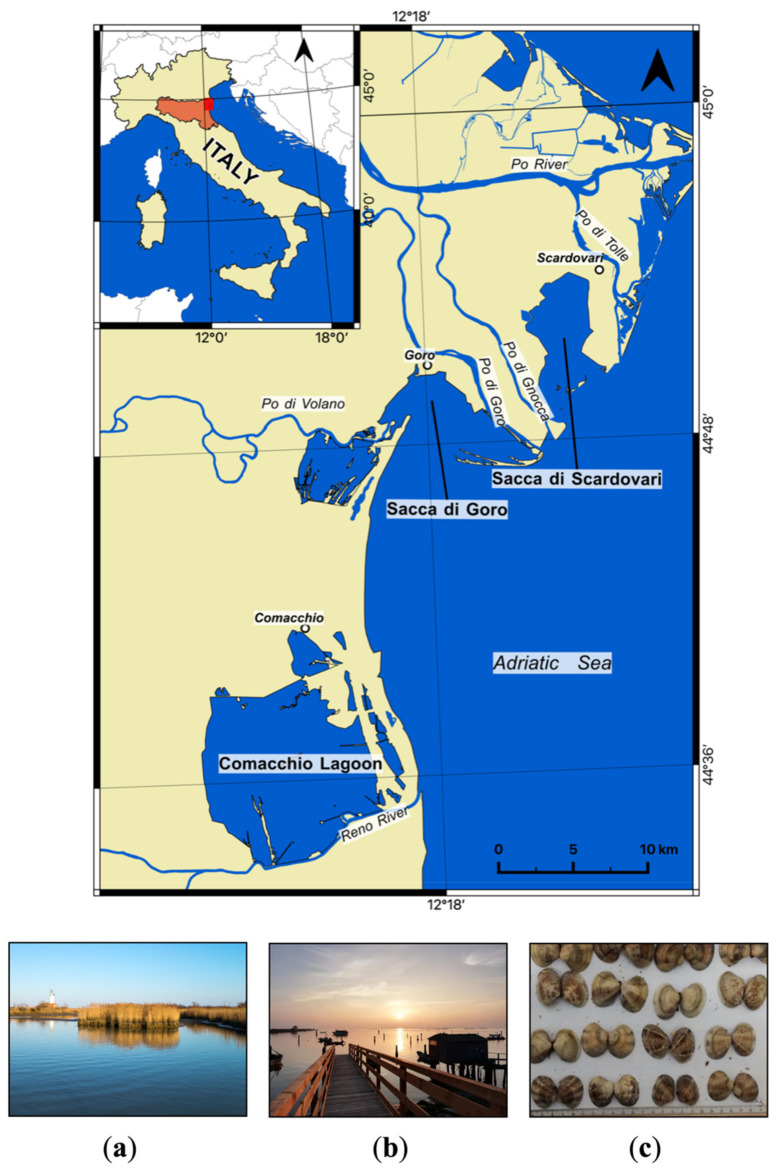
Geographic setting and locations of the investigated lagoons within the Po river delta area along the north-western Adriatic coast of the Emilia-Romagna and Veneto regions (Italy). Photos of (**a**) Sacca di Goro; (**b**) Sacca di Scardovari; and (**c**) Manila clams from Sacca di Goro.

The manila clam (*Ruditapes philippinarum*, Adams and Reeve, 1850) is a filter-feeding bivalve that lives buried in sandy mud sediments of lagoons, tidal flats and shallows. Today, manila clams are distributed worldwide, being one of the most important commercial shellfish species [1]. They are native to the Indo-Pacific waters and were introduced into Europe for aquaculture in the 1970s [2]. In 1983, the manila clam was introduced into the north-western Adriatic lagoons in Italy for the first time [3] (Figure 1) and now, the manila clam market represents a multi-million-euro business, supporting thousands of jobs in coastal communities. Recently, the Emilia-Romagna region (Northern Italy; Figure 1) aimed to register the manila clam habitat of Sacca di Goro, one of the most productive Adriatic lagoons, as a Protected Designation of Origin (PDO). The determination of clam authenticity is, therefore, essential to recognize clams with a misleading origin or adulteration, which (i) lower the product quality; (ii) contribute to illegal, unreported and unregulated (IUU) fishing; and (iii) may even constitute a risk to consumer’s health [4]. It follows that the ability to determine the origin of seafood is a powerful tool to protect both producers and consumers from potential fraud. Previous research proposed the use of genetic tools and multi-element analyses to identify the clam’s fingerprint [5,6,7]. However, there is still a need for the development of rapid, cost-effective tests that can shed light on the clam provenance to guarantee product quality and consumer safety.

Isotope ratio mass spectrometry (IRMS) is an automated, efficient and sensitive technique that is used for the identification, authenticity and traceability of materials. It is applied in different scientific fields, such as archaeology, biology, medicine and food authenticity [8,9,10,11,12,13,14]. The IRMS technique relies on the characteristic isotopic ratios of materials, which constitute unique signatures, as isotope enrichment or depletion processes depend on kinetic fractionation and environmental features [15]. Therefore, isotope signatures can be used to distinguish compounds by geographic origin that otherwise are chemically identical [8,11,15]. For example, carbon, nitrogen, sulfur, oxygen and hydrogen isotopic ratios measured with IRMS have been used to trace the geographical origins of a wide variety of products (e.g., wine, apple, pear, honey, potato, meat, rice [8,16,17,18,19,20,21,22,23]). Coherently, it is well known that a stable isotope composition of organic matrices, particularly “animal source foods”, reflects the environmental conditions of a product’s area of origin, the food web and the characteristic physiology of the organism [24]. Therefore, the investigation of isotopic variations of organism bodies (or part of them) represents a potential tool for their traceability and authentication.

Like other products, the provenance of seafood can be traced using the isotopic ratios of the organism tissues, as the carbon (C) and nitrogen (N) isotopic ratios are functions of the isotopic composition of the food source as well as the fractionation associated with biochemical processes [25,26,27,28]. However, Briant et al. [29] highlighted that temporal (from seasonal to multi-decadal) climatic variations can shift the isotope composition of mollusks. Seasonal variability has been related both to the reproduction cycle of bivalves and to changes in trophic resources, whereas multi-decadal climatic variations can modify the hydrological environment, thus affecting the biological or ecological responses of organisms. In addition, anthropogenic impacts can also alter the food web and the uptake of nutrients [28]. This, in turn, implies that isotope analyses represent potential tracers for the seafood origin but require the creation of datasets that must be updated periodically. In particular, manila clams feed exclusively on particles that are naturally present in the water column and on particles resuspended from the sediment [2,28]. Thus, stable isotopes could be used as a proxy to trace the provenance of these organisms, as there is a direct relationship between the isotopic composition of nutrients available in the in the ecosystem and the isotopic composition of consumer tissues [28,30,31,32,33]. Therefore, differences in isotopic values among manila clam tissues reflect a change of composition or shift in isotopic values of the relative food source [33], which can be used as fingerprint to trace the provenance of the organisms.

In this work, we evaluated the C and N isotopic ratios of whole clam tissues (i.e., foot, muscles, mantle and visceral mass) and the shells of manila clams as potential tools to be used in traceability in order to distinguish the organisms grown in selected Adriatic lagoons from those produced in other areas of the world, as well as to trace the exact Adriatic lagoon provenance for our samples. Such data were used to establish an archive that should be progressively updated to emphasize differences between the studied manila clams from north-western Adriatic lagoons and those from other productive areas, in order to identify possible frauds.

## 2. Materials and Methods

### 2.1. Study Area

This study was focused on three different lagoons located along the Italian northern Adriatic coast within the delta of the Po river, which is the principal Italian fluvial system [34,35]. In particular, we investigated clams from “Sacca di Goro” (44.82° N, 12.31° E) and “Sacca di Scardovari” (44.86° N, 12.42° E), two embayments connected to the Adriatic Sea through a wide mouth that are partly obstructed by sandy banks and are influenced by the interaction between fresh and marine waters that contributes to widespread deposit of the related sediment load [36,37]. Sacca di Goro is a micro-tidal lagoon (area: 26 km^2^; mean depth: 1 m; Figure 1a [36]) that receives nutrient-rich freshwater from two branches of the Po river, Po di Goro and Po di Volano (Figure 1). Sacca di Scardovari is a micro-tidal, large embayment (area: 32 km^2^; mean depth: 1.5 m; Figure 1b [36]) that receives nutrient-rich freshwater from other two branches of the Po river: Po di Gnocca and Po di Tolle (Figure 1). The third lagoon considered in this study was the Comacchio Lagoon (44.61° N, 12.17° E), which is a large non-tidal lagoon (area: 100 km^2^; mean depth: 0.8 m [36]) as it does not directly open toward the sea.

Shellfish farming in Sacca di Goro and Sacca di Scardovari occurs in zones at the boundary with open sea conditions and provides important socio-economic return [36,38]. On the contrary, in the Comacchio lagoon, although clams are observed among the benthic species present, shellfish farming is not so effective, because of the scarce oxygenation of water and organic matter-rich sediments.

### 2.2. Sample Collection and Pre-Treatment

Sampling of clams in the Sacca di Goro and Sacca di Scardovari was mainly carried out in May 2015 and May 2016 during site surveys focused on the study of sediments that were characterized by Natali and Bianchini [37]. For these samples the collection of clams was precisely georeferenced and sample numeration corresponded to that of the adjoining sediments studied by Natali and Bianchini [37]. Further samples from Sacca di Goro were provided by a local fisherman in June 2018 and were also bought from a local supermarket exhibiting certified provenance. A minor number of samples were collected from the Comacchio Lagoon in September 2017.

The shell lengths of the studied bivalves were measured (Figure 1c) and it was observed that they had sizes between 2 and 3 cm. Samples were dried at 60 °C and clam tissues were carefully separated from shells. The latter were washed (and brushed) with hydrogen peroxide and then dried again. Both shells and tissues were subsequently powdered with an agate mill.

### 2.3. Isotope Ratio Mass Spectrometry (IRMS)

The C and N elemental and isotope analyses were carried out at the Department of Physics and Earth Science of University of Ferrara (Italy) using an elemental analyzer (Vario Micro Cube, Elementar, Langenselbold, Germany) coupled with an isotopic ratio mass spectrometer (IsoPrime 100, Elementar, Manchester, UK) (Supplementary Figure S1) as proposed by Braint et al. [29] and McConnaughey and Gillikin [39], which analyze the C elemental and isotopic composition of mollusk tissues and shells, respectively.

Homogenous powdered samples (2–3 mg for clam shells, 5–10 mg for clam tissues) were weighed in tin capsules, wrapped and, finally, loaded in the Vario Micro Cube (Elementar, Langenselbold, Germany) autosampler to be analyzed. In this analytical system samples are burnt via flash burning with O_2_ at 950 °C in a quartz “combustion” tube. The N and C gaseous species released by the burnt samples are transferred in a second quartz “reduction” tube (heated at 550 °C), which reduces the nitrogen oxides (NO_x_) to N_2_. The formed N_2_ and CO_2_ are separated and the relative abundances are quantitatively determined on a thermo-conductivity detector. After the elemental analyses, N_2_ and CO_2_ flow in sequence to the coupled IRMS (Elementar, Manchester, UK) for isotopic composition determination. In the mass spectrometer the molecules of the sample gas are ionized by the source (i.e., a thorium oxide filament) and the ions pass through a magnet, which deflects and sorts them into beams with distinctive mass/charge ratios (*m*/*z*). Then, ion beams arrive at the collector where three Faraday cups detect the ions of each of the three different masses of analyzed gas simultaneously (i.e., for N_2_ the masses are 28, 29 and 30 and for CO_2_ the masses are 44, 45 and 46). In the cups, the impact of the ions is translated into a recordable electrical signal, forming peaks, with areas proportional to the number of incident ions. The isotope ratios are calculated through peak definition and integration.

Calibration of the instrument was performed using several standards: limestone JLs-1 [40], peach leaves NIST SRM1547 [41], caffeine IAEA-600, Jacupiranga carbonatite [42,43], Carrara Marble (cross-calibrated in a series of Italian laboratories) and synthetic sulfanilamide provided by Isoprime Ltd. Further details of the EA-IRMS technique are in the Supplementary Material.

^13^C/^12^C and ^15^N/^14^N isotopic ratios (R) were expressed in δ notation (in ‰ units):

(1) $$\delta =\left(\frac{R_{\mathrm{sam}}}{R_{\mathrm{std}}}-1\right)\times 1000$$

where R_sam_ is the isotopic ratio of the sample and R_std_ is the isotopic ratio of the international isotope standards Pee Dee Belemnite (PDB) and air N_2_, for C and N, respectively [44].

Analytical uncertainties (1 sigma) were in the order of ±0.1‰ and ±0.3‰ for C and N, respectively, as described by previous research [37,45,46,47,48].

### 2.4. Statistical Analysis

The statistical analysis was conducted in R [49]. The analysis of variance (ANOVA test) was applied to every variable in order to determine the statistical differences between clams from distinct lagoons. Pairwise comparisons were performed using Tukey’s HSD (honestly significantly different) test. Principal component analysis (PCA) was applied to examine differences in elemental and isotopic parameters between manila clam tissue samples of different origin (package “FactoMineR” [50]; package “factoextra” [51]).

## 3. Results

**Figure 2:**
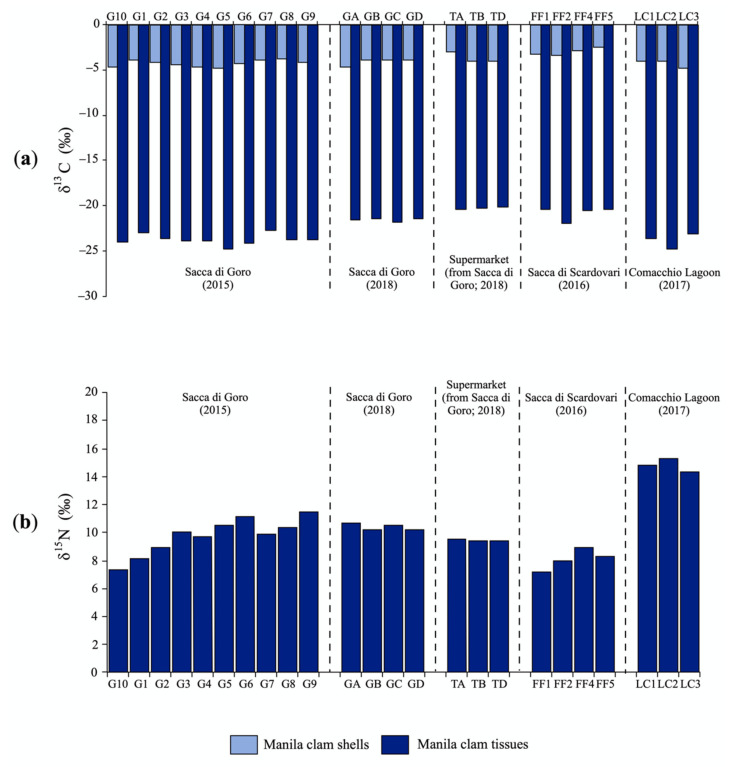
(**a**) Carbon (C) isotopic composition of carbon of shells and tissues and (**b**) nitrogen (N) isotopic composition of tissues of manila clam samples collected onsite from north-western Adriatic lagoons and bought at the local supermarket.

The contents of C and N and the respective isotopic ratios of the manila clam shells and tissues are reported in Table 1 and Figure 2.

**Figure 3:**
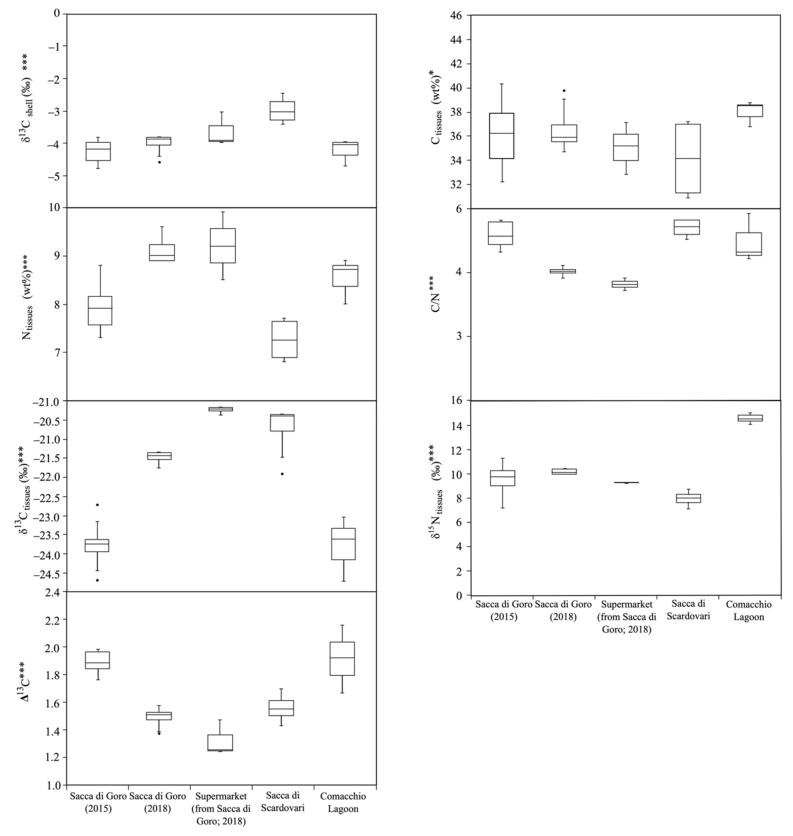
Box plot of elemental and isotopic compositions of carbon (C) and nitrogen (N) in the shells and tissues of manila clam samples collected onsite from north-western Adriatic lagoons and bought at the local supermarket. One-way ANOVA is also reported (* *p* < 1; ***p < 0.0001).

The one-way ANOVA test showed that most elemental and isotopic variables of manila clams were highly affected by the geographical area and time of sampling (*p*-values < 0.0001), with the only exception of tissues C content (*p*-value < 1) (Figure 3).

The C isotopic values of aragonite shells of sampled clams were similar: samples collected onsite from Sacca di Goro are similar to those of Comacchio, but they were more negative than those from Sacca di Scardovari and those from the supermarket, despite having the same provenance.

Considering the manila clam tissues, the C and N contents varied among the samples without defined inter- or intra-lagoon trends. For all manila clam samples, the C/N ratio was ~4, which is indicative of the similar lipid contents, which is in turn correlated with the size of the bivalve [2,52].

The δ^13^C values of tissues were homogeneous among the clams collected from the same lagoons: δ^13^C values of Sacca di Goro clams sampled in 2015 and Comacchio Lagoon clams are similar and they were more negative than those from Sacca di Goro sampled in 2018, the supermarket, and Sacca di Scardovari (Table 1; Figure 2a).

The δ^15^N ratio values were uniform among samples with the same origin, but there were significant variations among bivalves from different lagoons to another. The clams with less positive values were from Sacca di Goro and Sacca di Scardovari (Table 1; Figure 2b), whereas the most positive values were recorded for samples from Comacchio Lagoon (Table 1; Figure 2b). This was probably related to persistent oxygen depletion in this area, as the circulation of water is limited.

## 4. Discussion

### 4.1. δ13. C vs. δ^15^N Binary Diagram: An Approach to the Seafood Traceability at the Regional Scale

**Figure 4:**
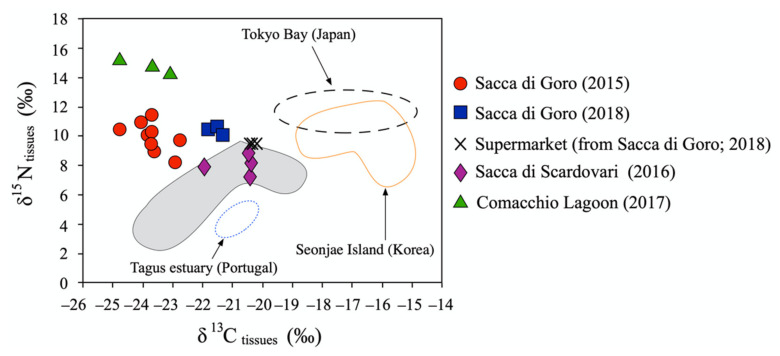
Comparison of carbon (C) and nitrogen (N) isotopic compositions of manila clam tissues from northern Adriatic lagoons with those from fisheries worldwide (Tagus estuary, Portugal [2]; Seonjae Island, Korea [52]; Tokyo Bay, Japan [56]). The grey area represents the C-N isotopic composition of Po suspended solids (data from [57,58]).

The related body of literature shows that the C isotopic ratio of seafood [2,24,53] has routinely been utilized for the identification of food sources, whereas the N isotopic ratio provides information about the circulation of water and nutrients, as less positive values are associated with better exchange of water in the environment [54,55]. Thus, the δ^13^C-δ^15^N composition of the samples reflect the characteristics of their area of origin and could be used to trace the provenance of organisms. In fact, in Figure 4, a simple binary diagram δ^13^C vs. δ^15^N is able to distinguish the different provenances of the investigated manila clams at least at the regional scale.

In general, the δ^13^C values of Adriatic manila clam samples resemble that of Po river suspended solids analyzed by Tesi et al. [57] and Corazzari et al. [58]. Determination of the δ^13^C-δ^15^N composition of Adriatic manila clams is useful to depict the “state of health” of the lagoons during the sampling survey. In fact, considering the samples collected onsite in lagoons, less negative δ^13^C and less positive δ^15^N tissues are typical of Sacca di Scardovari, where the circulation of freshwater and/or nutrients is limited. On the contrary, very positive δ^15^N ratios coupled with very negative δ^13^C ratios are characteristic of Comacchio Lagoon clams, as the lagoon is poorly connected with the sea, limiting the exchange of water and causing consistent input of nutrients from the agricultural run-off [36]. The composition of Sacca di Goro samples is between those of Sacca di Scardovari and the Comacchio Lagoon. For the Sacca di Goro samples, the isotopic composition varied year-by-year, demonstrating that climate and environmental changes also play significant roles in determining on the isotopic compositions of the organisms. In addition, the Sacca di Goro samples obtained from the market had the least negative δ^13^C values, as before selling, clams expel sediment and nutrients in ranks of saline water, where they continue their filtering activity and consequently, biological fractionation proceeds.

### 4.2. Multivariate Analyses for the Seafood Traceability at the Local Scale

It is clear that a bivariate analysis is not sufficient for tracking the geographical origin of manila clam samples in a restricted area. Therefore, more parameters must be considered to constrain the provenance of these products. For example, shells are generally excluded when assessing the traceability of manila clams, as the isotopic values of shell carbonates of marine and freshwater mollusks reflect the δ^13^C isotopic value of the dissolved inorganic carbon, not the dietary resources [32]. However, according to the results obtained in a feeding experiment conducted by Poulain et al. [59] on *Ruditapes philippinarum*, the δ^13^C of shell is affected by both the HCO_3_^–^ content in seawater and the diet of the bivalve. In fact, in this study, the isotopic C values of manila clam shells were shifted toward negative δ^13^C values, as they incorporated isotopically light metabolic C. Therefore, in our view, the δ^13^C of shells can be also used as index of the seafood provenance, as this isotopic ratio could reflect the medium-long term isotopic growth of these low motile species.

Thus, to trace the geographical provenance of the samples, we conducted a principal component analysis (PCA) in order to group the clams on the basis of many variables called principal components, which can describe correlations among the studied samples. As principal components, we considered the δ^13^C and δ^15^N of tissues, the δ^13^C of shells, C/N, as well as ∆^13^C, a new parameter calculated as the difference between the δ^13^C of shells and δ^13^C of tissues. Validation of the model was obtained by cross-validation (CV) using the leave-one-out procedure (LOO).

**Figure 5:**
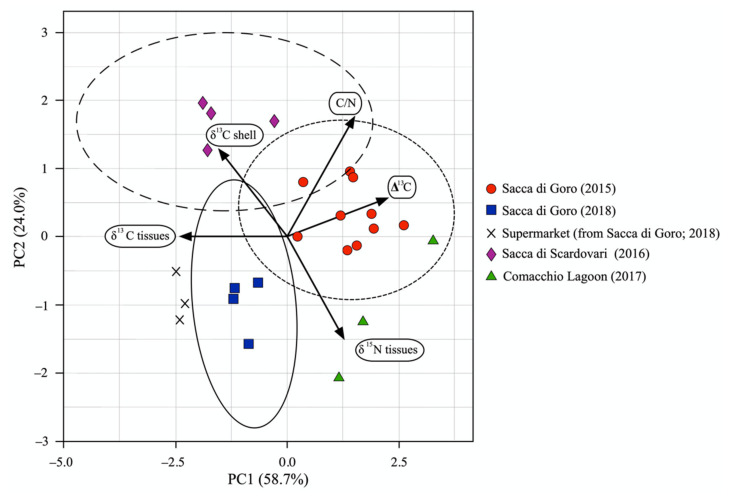
Principal component analysis (PCA) for the δ^13^C of shells, δ^13^C, δ^15^N and C/N of tissues and ∆^13^C in manila clams collected onsite from north-western Adriatic lagoons and bought at a local supermarket.

The resulting PCA (Figure 5) explains more than 80% of the total variation and well clusters the samples into four groups reflecting their different geographical origins and, in the case of Sacca di Goro, also the year in which clams were collected. Sacca di Scardovari and Comacchio Lagoon samples formed distinct groups, with Sacca di Scardovari samples being characterized by less negative δ^13^C values for both shells and tissues and the Comacchio Lagoon group was driven by high δ^15^N and more negative δ^13^C values of tissues. For Sacca di Goro, samples collected in 2015 were separated from those collected in 2018, mainly in terms of the ∆^13^C and C/N values. Interestingly, the clams bought at the supermarket, with the label certifying they were collected in Sacca di Goro in 2018, were plotted near to the group of samples collected onsite in Sacca di Goro in the same year, as they are characterized by similar values in δ^13^C of tissues and shell, C/N and ∆^13^C. Contrary to the binary plot shown in Figure 4, the PCA plot of Figure 5 clearly shows that the certified manila clams bought at the supermarket were similar to the samples collected onsite in Sacca di Goro in 2018. This demonstrates that a multivariate analysis is the best approach for tracing clams at the regional scale, as it condenses a large number of original variables into a composite dimension with a minimum loss of information.

On the other hand, at the world scale, the C and N isotopic compositions of manila clams seem to be sufficient for the identification of at least the country of the origin. In fact, in Figure 4 the entire manila clam population from the north-western Adriatic lagoons is shown to be isotopically distinct from those of other *Ruditapes philippinarum* populations growing in other fisheries widespread throughout the world (e.g., Portugal, Korea, Japan [2,52,59]), as it has the most negative δ^13^C (up to –24.7‰) value and the most positive δ^15^N (up to +15.2‰) value. Therefore, at a large scale, the diet of bivalves becomes the main variable for the isotopic characterization of the clams. Generally, filter-feeding bivalves feed on marine particulate organic matter suspended in the water column (e.g., phytoplankton, microphytobenthos and detritus [36]). In the north-western Adriatic lagoons, the dispersed particulate is deeply affected by the fluvial contribution of the Po river system that conveys a large amount of solid suspended load with δ^13^C and δ^15^N signatures up to −28.2‰ and +8.6‰, respectively, toward the sea [57,58]. In fact, as shown in Figure 4, considering the trophic enrichments of *Ruditapes philippinarum* (0.6‰ for δ^13^C and 3.4‰ for δ^15^N; [32]), the isotopic compositions of the tissues of the investigated clams can easily be related to the average δ^13^C and δ^15^N signatures of the Po river suspended load.

## 5. Conclusions

Food safety is generally monitored through protocols involving physicochemical or biological parameters that are compared with tolerance thresholds, but these analyses often do not guarantee the identification of the specific geographic origins of the considered products. The certification of the area of provenance of food products is difficult to constrain, but it is needed to increase the consumer confidence. In fact, consumers want to make an informed choice to have genuine products for both health and ecological reasons and therefore, products have to be traceable and authenticated. Scientific tools for food traceability are crucial to avoid product mislabeling and fraudulent activities, which sometimes occur due to increasing demand, resource limitations, or the high value of the original supply chain. Therefore, product differentiation appears to be a fundamental issue in the further development of the fish farming industry widespread in different rearing and environmental systems across the whole Mediterranean area.

In this framework, the presented research emphasizes that IRMS is a promising technique for tracking the geographic origin of mollusks, in particular, manila clams from the north-western Adriatic lagoons, which are affected by counterfeiting with clams of exotic and dubious provenance. The IRMS has many applications in several fields including geology, biology, ecology and, more recently, food sciences, thanks to the high levels of accuracy and precision that can be reached and the ability to analyze low masses of material without sample pre-treatment.

The isotopic composition of clams obviously reflects that of the ecosystem in which they live, their trophic level and the food web. Manila clams from selected north-western Adriatic lagoons live in an aquatic environment that is rich in nutrients and characterized by low (very negative) δ^13^C and high δ^15^N values. This isotopic fingerprint of tissues, although slightly modified by biological fractionation, is transferred to local clams, which, on this basis, can be distinguished from products that have originated elsewhere in the world. More difficult is the identification of the precise point of origin of different manila clams produced in nearby lagoons with similar environmental and climatic conditions. In this case, the use of more variables (e.g., isotopic fingerprint of shells and C/N as well as the isotopic composition of tissues) is requested to trace the sample provenance in more detail.

We are aware that C and N isotopic shifts could be partly related to the different climatic conditions that possibly occurred during the diachronous sampling survey conducted in this study. For this reason, further research should involve the analysis of a larger number of samples, possibly collected simultaneously in the distinct north-western Adriatic lagoons, with the aim of creating a more robust isotopic archive to compare unknown samples with referenced datasets. Moreover, additional isotopic tracers should be taken into account to corroborate the robustness of the hypothesis. In this light, our research will continue, in order to (1) analyze further manila clams from the considered lagoons to validate the outlined δ^13^C-δ^15^N compositional range and (2) to explore other isotopic parameters (e.g., sulfur and oxygen isotope analyses) measurable by IRMS that could be useful as a proxy of their origin and provenance.

## Figures and Tables

**Table 1:** Average elemental and isotopic compositions of carbon (C) and nitrogen (N) in the shells and tissues of manila clam samples collected onsite from north-western Adriatic lagoons and bought at the local supermarket. Δ^13^C was calculated as the difference between the δ^13^C of shell and tissues of the clam samples. Means followed by the same letter did not differ significantly (Tukey’s HSD, *p* > 0.05).

Adriatic lagoons	Samples	Shell	Tissues					Δ^13^C
		δ^13^C (‰)	C (wt%)	N (wt%)	C/N	δ^13^C (‰)	δ^15^N (‰)	
Sacca di Goro (2015)	10	–4.2 ± 0.3 ^a^	36.1 ± 2.7 ^a^	7.9 ± 0.4 ^ab^	4.6 ± 0.2 ^b^	–23.7 ± 0.6 ^a^	9.7 ± 1.3 ^ab^	19.5 ± 0.4 ^b^
Sacca di Goro (2018)	4	–4.0 ± 0.4 ^a^	36.6 ± 2.2 ^a^	9.1 ± 0.3 ^c^	4.0 ± 0.1 ^a^	–21.5 ± 0.2 ^b^	10.3 ± 0.2 ^b^	17.5 ± 0.4 ^a^
Supermarket (from Sacca di Goro; 2018)	3	–3.6 ± 0.5 ^ab^	35.0 ± 2.1 ^a^	9.2 ± 0.7 ^c^	3.8 ± 0.1 ^a^	–20.2 ± 0.1 ^b^	9.4 ± 0.1 ^ab^	16.6 ± 0.6 ^a^
Sacca di Scardovari (2016)	4	–3.0 ± 0.4 ^b^	34.1 ± 3.4 ^a^	7.2 ± 0.5 ^a^	4.7 ± 0.2 ^b^	–20.8 ± 0.8 ^b^	8.0 ± 0.7 ^a^	17.8 ± 0.6 ^a^
Comacchio Lagoon (2017)	3	–4.2 ± 0.4 ^a^	38.0 ± 1.0 ^a^	8.5 ± 0.5 ^bc^	4.5 ± 0.3 ^b^	–23.8 ± 0.9 ^b^	14.7 ± 0.5 ^c^	19.6 ± 1.2 ^b^

## Data Availability

Not applicable.

## Supplementary Materials

The following are available online, Supplementary material: Details of EA-IRMS technique; and Figure S1: (a) Photos and (b) configuration of elemental analyzer (EA, Vario Micro Cube, Elementar, Langenselbold, Germany) in line with an isotope ratio mass spectrometer (IRMS, IsoPrime 100, Elementar, Manchester, UK) of the Department of Physics and Earth Science of University of Ferrara (Italy).
