# Supplementary material for: Traceability and Authentication of Manila Clams from North-Western Adriatic Lagoons Using C and N Stable Isotope Analysis
Source: Molecules. 2021 Mar 25;26(7):1859. doi: 10.3390/molecules26071859 (PMC8037060; doi:10.3390/molecules26071859)
Supplement: Supplementary file 1 [file molecules-26-01859-s001.pdf]

Supplementary material

Material and Methods

Details of the Isotope Ratio Mass Spectrometry (IRMS)

The C and N elemental and isotope analyses of manila clam shells and tissues were carried out at the Department of Physics and Earth Science of University of Ferrara (Italy) using an elemental analyzer (Vario Micro Cube, Elementar, Langenselbold, Germany) coupled with an isotopic ratio mass spectrometer (IsoPrime 100, Elementar, Manchester, UK) (Supplementary Figure S1).

Homogenous powdered samples (2-3 mg for clam shells, 5-10 mg for clam tissues) are weighed in tin capsules, wrapped, and finally loaded in the Vario Micro Cube autosampler to be analyzed. Flash burning occurs at 950 °C in a quartz "combustion" tube filled with copper oxide chips (sealed on both sides by corundum balls and quartz wool) which function as catalyst in presence of high purity (6 grade purity) O_2 gas. The C and N gaseous species released by the burnt samples are transferred in a second quartz "reduction" tube (heated at 550 °C) that contains chips of native copper to reduce the nitrogen oxides (NO_x) to N_2. The formed CO_2 and N_2, carried by dry He (5 grade purity) gas, flow within a drying tube containing Sicapent® to remove H_2O, and then are separated by a temperature programmable desorption column (TPD), and quantitatively determined on a thermo-conductivity detector (TCD). Nitrogen (N_2) flow first to the coupled IRMS for isotopic composition determination, while CO_2 is sequestrated in the TPD column, kept at room temperatures 20–25 °C. When the N isotopic analysis terminated, CO_2 is desorbed from the TPD, increasing the temperature to 210 °C and finally conveys the IRMS for the measurement of C isotopic ratios. In this case, before to enter the IRMS, the sample gas is diluted with additional He, as most organic compounds contain relatively larger proportion in C rather in N, and hence the CO_2 signal must be diluted to attain an appropriate signal. The mass spectrometer configuration comprises an ionization source, a flight tube, a magnet, and a series of collectors. In the instrument the molecules of the sample gas are ionized by the source (i.e., a thorium oxide filament) and the resulting ions are accelerated by an electric field. The ions move from the source into a curved flight tube passing through a magnet, which deflects and sorts them into beams having distinctive mass/charge ratio (m/z). Then ion beams pass through a resolving slit into the collector, where three Faradays cups detect simultaneously the ions of each of the three different masses of the analyzed gas (e.g., for N_2 the masses are 28, 29, and 30 and for CO_2 the masses are 44, 45, and 46). The detection of the distinct isotopic masses of the sample is bracketed between those of reference gases (N_2 and CO_2, 5 grade purity) gases, which have been calibrated using reference materials. In the cups, the impact of the ions is translated into a recordable electrical signal, forming peaks, which area is proportional to the number of incident ions. The isotope ratios are calculated through peak definition and integration. The signal intensity is amplified by an integrated Amplifier and is expressed in nanoampere (nA). The signal intensity is referred as "peak height", the minimum acceptable signal is 1 nA (optimum between 2 and 10 nA) in amplitude and at least 5 seconds in duration.

Calibration of the instrument was performed using several standards: limestone JLs-1 [40], peach leaves NIST SRM1547 [41], caffeine IAEA-600, Jacupiranga carbonatite [42,43], Carrara Marble (cross-calibrated in a series of Italian laboratories), and synthetic sulfanilamide provided by Isoprime Ltd.

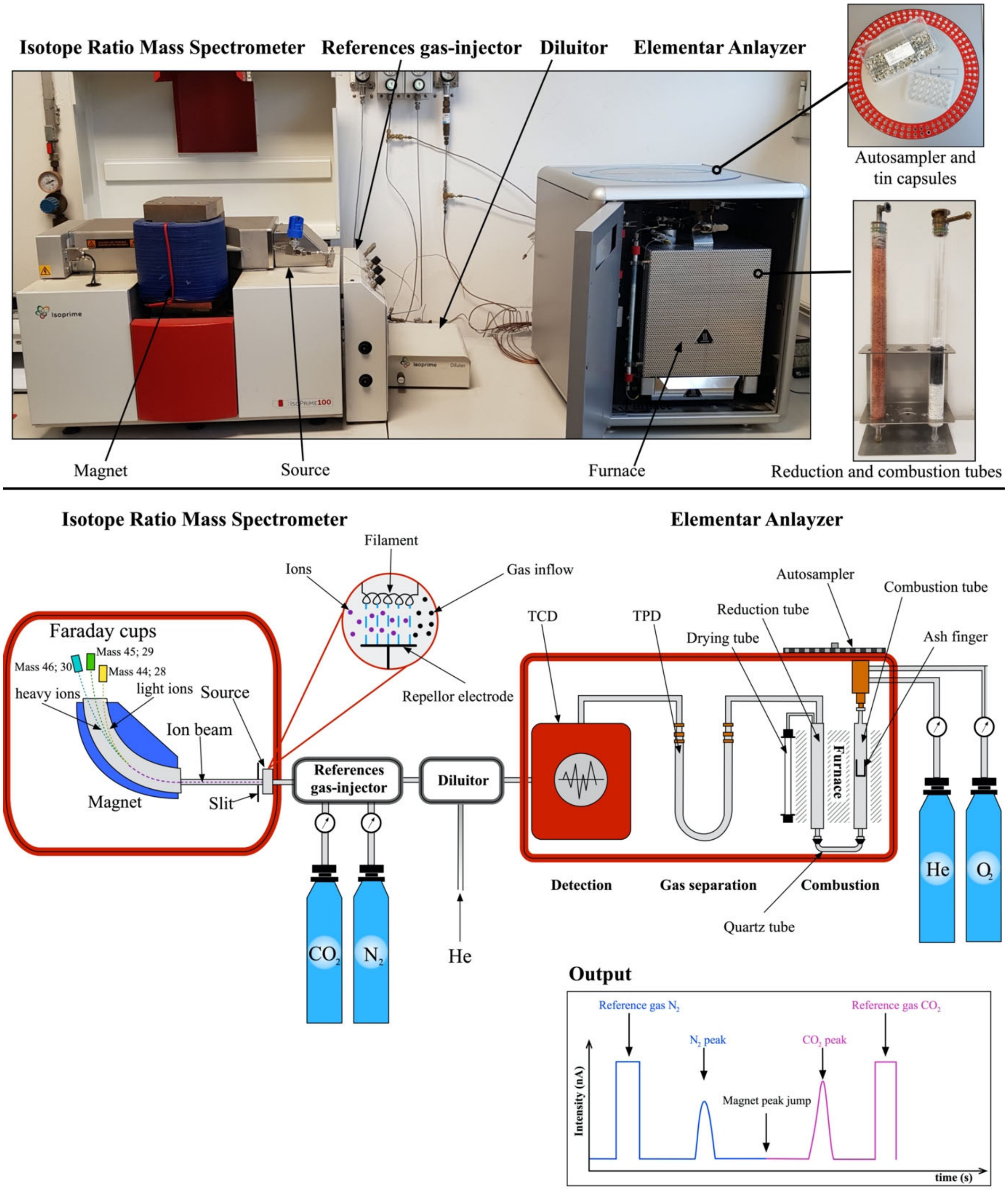

Figure S1. (a) Photos and (b) configuration of elemental analyzer (EA, Vario Micro Cube, Elementar, Langenselbold, Germany) in line with an isotope ratio mass spectrometer (IRMS, IsoPrime 100, Elementar, Manchester, UK) of the Department of Physics and Earth Science of University of Fer-rara (Italy).
